# Cross-Sectional Assessment of Dermatologists’ Knowledge and Attitude Towards Isotretinoin-Related Ocular Side Effects in Aseer, Saudi Arabia

**DOI:** 10.7759/cureus.46335

**Published:** 2023-10-01

**Authors:** Abdullah Korkoman, Abdulrahman Alamri, Ahmed S AL Zomia, Sultana Korkoman, Saad H Qahtani, Yazeed M Alshahrani, Turki B Alotaibi, Bandar M Asiri, Abdulrahman N Alqahtani, Omar A Awwadh, Bader A Alghamdi, Abdulaziz Alshahrani

**Affiliations:** 1 Medicine, College of Medicine, King Khalid University, Abha, SAU; 2 Ophthalmology, College of Medicine, King Khalid University, Abha, SAU; 3 Medicine and Surgery, Bisha University, Bisha, SAU; 4 General Medicine, College of Medicine, King Khalid University, Abha, SAU; 5 College of Medicine, Bisha University, Bisha, SAU; 6 Family Medicine, King Khalid University Hospital, Abha, SAU; 7 General Medicine, Asser Central Hospital, Abha, SAU; 8 Family Medicine, King Fahad Military Medical Complex, Abha, SAU

**Keywords:** dermatologists, ocular side effects, saudi arabia, acne vulgaris, isotretinoin

## Abstract

Background: Acne vulgaris is a chronic inflammatory disease of the pilosebaceous unit, which includes the hair follicle, hair shaft and sebaceous gland. The only treatment that has an effect on all the main aetiological causes of acne is isotretinoin. However, it may have a variety of negative side effects. The aim of this study was to evaluate the knowledge and attitudes of dermatologists regarding ocular effects following isotretinoin prescribing in the Aseer region, Saudi Arabia.

Methodology: An anonymous online cross-sectional survey was conducted in August 2022 to investigate dermatologists' knowledge and attitude regarding isotretinoin-related ocular side effects in Aseer, Saudi Arabia. Participants were recruited using convenience and snowball sampling methods.

Results: A total of 48 dermatologists were included in this survey. The age distribution ranged from 25 to over 60 years. Sixteen (37.5%) were aged 31-40 years, 29 (60.4%) were male and 20 (41.7%) were specialists. All dermatologists reported that it can cause dry eye, 32 (66.7%) reported that it can cause contact lens intolerance, 10 (20.8%) reported that it can cause a decrease in dark adaptation and two (4.2%) thought that it can cause ectopia lentis and retinoblastoma. Regarding attitude, 43 (87.5%) thought that a course of isotretinoin is not recommended if the patient has recently undergone refractive surgery, 30 (62.5%) always inform patients about ocular side effects, 31 (72.9%) do not consider referring patients for ophthalmic examinations before initiating isotretinoin, 12 (25.0%) always prescribe lubricant eye drops, 15 (31.3%) always ask patients about recent refractive surgery, 17 (35.4%) always warn patients about avoiding refractive surgery during isotretinoin use and 19 (39.6%) always inform patients concerning discomfort with contact lenses.

Conclusions: The survey reveals that dermatologists in Aseer, Saudi Arabia, generally have good knowledge of isotretinoin related to some ocular side effects, especially dry eye and contact lens intolerance. However, there is some variation in their practices with regard to patient education, referrals for ophthalmic examinations, the use of lubricant eye drops and refractive surgery during isotretinoin treatment. Thus, dermatologists should receive educational training on the safety profile of isotretinoin while managing acne vulgaris.

## Introduction

Acne vulgaris is one of the most widespread dermatological diseases in the world, affecting an estimated 650 million people. It is a chronic inflammatory disease of the pilosebaceous unit, which includes the hair follicle, hair shaft and sebaceous gland [1,2]. Due to its prolonged course, pattern of recurrence/relapse and signs such as severe outbreaks or delayed onset, acne is regarded as a chronic disease. Additionally, acne has a significant detrimental psychological and social impact on a patient's quality of life [3]. More than 95% of adolescents and 85% of adults suffer from acne at some point in their lives [3,4]. Almost 20% of these adolescents had moderate to severe acne, and up to 50% of them still experience acne as adults [5]. Acne ranked seventh internationally in terms of prevalence according to a comprehensive examination of the Global Burden of Disease study in 2010 [6]. Numerous processes are involved when acne forms in the pilosebaceous unit. Disrupted sebaceous gland activity linked to hyperseborrhea and changes in the composition of fatty acids in sebum, dysregulation of the hormone microenvironment, interaction with neuropeptides, follicular hyperkeratinization, inflammation induction and dysfunction of the innate and adaptive immune systems are just a few of the key processes underlying acne development [7]. Due to impairment of the pilosebaceous unit by these processes, normal pores develop into microcomedones, which then develop into comedones and inflammatory lesions. Antigens from bacteria can make the inflammation worse [8,9]. Other variables that might cause or aggravate acne include UV radiation and other environmental factors [10,11], dietary factors [12], smoking [13], stress and modern lifestyle [14].

The only treatment that has an effect on all of the main aetiological causes of acne is isotretinoin, which affects cell-cycle development, cellular differentiation, cell survival and apoptosis in order to accomplish this astounding efficacy [15]. Although isotretinoin may have a variety of negative side effects, serious effects are uncommon [16,17]. Depression is one of the negative consequences, albeit rare, that should be looked out for [18,19]. With regard to isotretinoin’s teratogenicity, the quality-adjusted life years (QALY) benefit of treating patients with severe, moderate and mild acne is 50, 11 and seven times greater, respectively, than the risk of loss of QALY from isotretinoin teratogenicity, according to an estimate of the expected risk. This is despite the fact that isotretinoin may cause severe birth defects [18].

The Food and Drug Administration approved isotretinoin for moderate and severe acne but dermatologists have misused it for mild cases, thus increasing the risk of adverse effects on the ocular system. Neudorfer et al. [20] found a strong association between isotretinoin therapy and clinically meaningful ocular adverse events, with the peak increased risk occurring four months after the first prescription. Common adverse events include conjunctivitis, hordeolum, chalazion, blepharitis, eye pain and dry eye, with acute conjunctivitis being the most frequent diagnosis.

A cross-sectional study in Egypt found that 94.6% of dermatologists were aware of ocular side effects, 91.1% informed patients about the drug's adverse effects and 74.8% knew about contact lens intolerance. Most dermatologists routinely prescribed lubricant eye drops and 56.8% referred patients to an ophthalmologist [21]. This study’s hypothesis is that there is a lack of sufficient knowledge about the ocular side effects of isotretinoin among dermatologists in Saudi Arabia. The aim of this study was to evaluate the knowledge and attitudes of dermatologists regarding ocular effects following isotretinoin prescribing in the Aseer region, Saudi Arabia.

## Materials and methods

Study design and settings

An anonymous online cross-sectional survey was conducted in September and October of 2022 to investigate dermatologists' knowledge and attitude regarding isotretinoin-related ocular side effects in Aseer, Saudi Arabia. Participants were recruited through convenience and snowball sampling methods.

Sample size and study population

Using G *Power 3.1, assuming an effect size of 0.1, an alpha error of 5%, and a power of 80%, the minimum required sample size to detect differences in knowledge and attitude related to isotretinoin-related ocular side effects was found to be 199. To account for a potential non-response rate of 30%, we increased the sample size to 300. Dermatologists practicing in Aseer, Saudi Arabia, regardless of their subspecialty, were invited to participate in this study. Participants were required to have internet access through smartphones, tablets, or computers.

Study outcomes

The primary objective of this study was to assess the knowledge and attitude of dermatologists in Aseer, Saudi Arabia, regarding isotretinoin-related ocular side effects. 

Data collection

The survey questionnaire was meticulously developed by incorporating insights from a literature review and relevant questions from similar studies in the field. Content and face validity tests were performed to validate the questionnaire. Content validity involved expert feedback and domain experts' insights to assess the relevance, clarity, and comprehensiveness of the questions, leading to necessary adjustments. Face validity testing included piloting the questionnaire with a small sample to gauge participants' understanding and identify any ambiguities. Feedback from the pilot study refined the questionnaire's clarity and suitability. The internal consistency of the tool was evaluated using Cronbach's alpha, resulting in a high coefficient of 0.83, indicating strong consistency among the questionnaire items. The questionnaire was uploaded to a Google form and distributed through commonly used social media used in Saudi Arabia like Telegram, Facebook, and Messenger. Each data collector sent the questionnaire to a sample of people during the pilot research to check that it was completed quickly and clearly. According to the pilot study, the completion time was between five and nine minutes, with some small alterations made for clarity. The final analysis did not contain any of the data from the pilot study. One response per participant was guaranteed thanks to measures taken to stop duplicate submissions.

Ethical approval

Saudi Arabia's King Khalid University Ethics Committee approved this study (ECM 2023-2301). Participants gave their permission to participate at the outset of the survey, giving them the freedom to choose whether to give their permission and fill out the survey or not. All personal data collected from participants throughout the study were kept in tight confidence and anonymous, according to the research team.

Statistical analysis

Statistical analysis was performed using R software version 4.3.1 (R Foundation for Statistical Computing, Vienna, Austria). Categorical variables were presented as numbers and percentages. Office 2016 (Microsoft, Redmond, WA, USA) was used to create the figures.

## Results

Table 1 presents the demographic characteristics of the study participants. The age distribution ranged from 25 to over 60 years old, with nearly one-third (37.5%, n=18) aged 31-40 years. The gender distribution shows a higher representation of male dermatologists (60.4%, n=29) compared to female dermatologists (39.6%, n=19). A substantial proportion held the title of consultant (39.6%, n=19) and a significant number were classified as specialists (41.7%, n=20). Additionally, the representation of resident dermatologists was nine (18.8%) and for those associated with military hospitals it was six (12.5%).

**Table 1:** Demographic characteristics of the study participants

Overall (N = 48)		N (%)
Age	25–30 years	9 (18.8%)
	31–40 years	18 (37.5%)
	41–50 years	13 (27.1%)
	51–60 years	6 (12.5%)
	Older than 60 years	2 (4.2%)
Gender	Female	19 (39.6%)
	Male	29 (60.4%)
Qualification	Consultant	19 (39.6%)
	Resident	9 (18.8%)
	Specialist	20 (41.7%)
	Military hospital	6 (12.5%)
Health Institution	Ministry of Health hospital	25 (52.1%)
	Private clinic	14 (29.2%)
	University	3 (6.3%)

**Figure 1:**
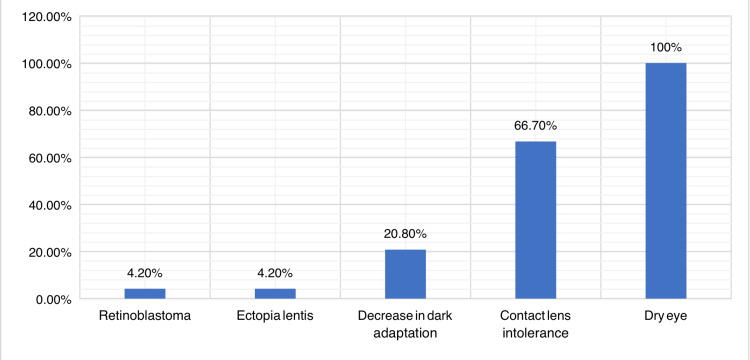
Dermatologists’ knowledge about the ocular side effects associated with isotretinoin for treating acne vulgaris.

Figure 1 shows the perceived side effects of isotretinoin by dermatologists in the Aseer region. All dermatologists reported that it can cause dry eye, two-thirds (66.7%, n=39) reported that it can cause contact lens intolerance and one-fifth reported that it can cause a decrease in dark adaptation. Finally, two (4.2%) thought that it can cause ectopia lentis and retinoblastoma.

**Figure 2:**
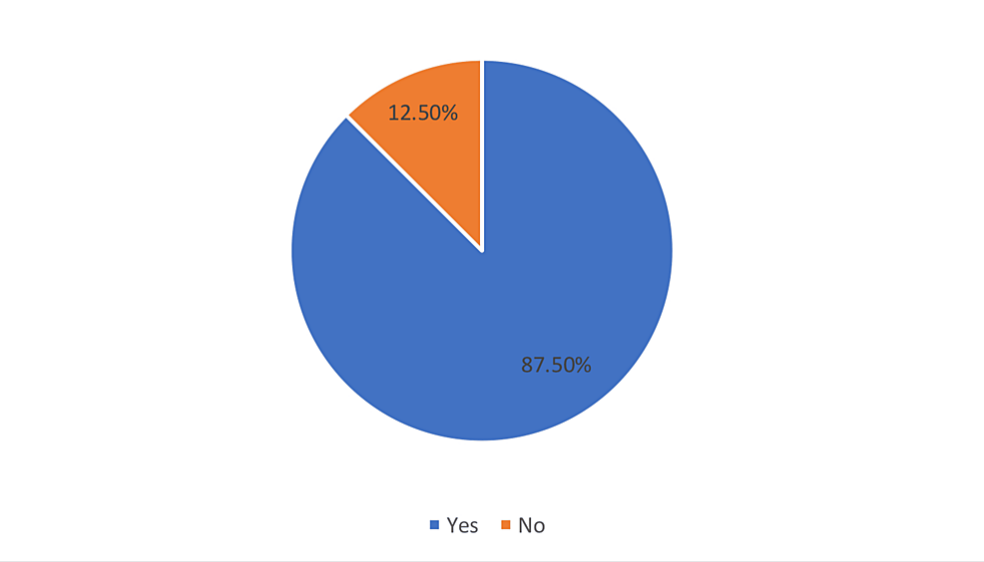
Starting an isotretinoin course is not recommended if the patient has recently undergone refractive surgery

The majority (87.5%) of dermatologists surveyed thought that isotretinoin course is not recommended if the patient had recently undergone refractive surgery (Figure 2).

Table 2 provides numerical data and corresponding percentages concerning healthcare professionals' practices in relation to ocular side effects and patient care associated with isotretinoin: 62.5% (n=30) of professionals always inform patients about ocular side effects, 31.3% (n=15) do so sometimes and only 6.3% (n=3) rarely do so. A significant majority (72.9%, n=32) do not consider referring patients for ophthalmic examinations before initiating isotretinoin, with only 27.1% (n=13) believing that it is important. There is a range in the frequency of referrals to ophthalmologists, with 43.8% (n=21) never referring, 37.5% (n=18) rarely referring and 18.8% (n=9) sometimes referring. In terms of prescribing lubricant eye drops, 56.3% (n=27) do so sometimes, 25.0% (n=12) always, 12.5% (n=6) rarely and 6.3% (n=3) never. Asking patients about recent refractive surgery varies, with 31.3% (n=15) always, 29.2% (n=14) rarely, 25.0% (n=12) sometimes and 14.6% (n=7) never inquiring. Warnings about avoiding refractive surgery during isotretinoin use vary from 35.4% (n=17) always, 29.2% (n=14) rarely, 20.8% (n=10) sometimes and 14.6% (n=7) never providing such warnings. Concerning discomfort with contact lenses, 39.6% (n=19) always inform patients, 33.3% (n=12) sometimes, 14.6% (n=7) rarely and 12.5% (n=6) never provide this information.

**Table 2:** Survey of healthcare professionals' practices and awareness regarding isotretinoin-related ocular side effects

Question	Response	N (%)	
Do you inform your patients about the ocular side effects of isotretinoin	Always	30 (62.5%)	
	Sometimes	15 (31.3%)	
	Rarely	3 (6.3%)	
Do you think it is important to refer the patients to do an ophthalmic exam before starting isotretinoin course	No	35 (72.9%)	
	Yes	13 (27.1%)	
How often do you refer to an ophthalmologist before starting isotretinoin course	Never	21 (43.8%)	
	Rarely	18 (37.5%)	
	Sometimes	9 (18.8%)	
Do you prescribe lubricant eye drops with isotretinoin	Always	12 (25.0%)	
	Never	3 (6.3%)	
	Rarely	6 (12.5%)	
	Sometimes	27 (56.3%)	
Do you ask patients about recent refractive surgery before starting isotretinoin	Always	15 (31.3%)	
	Never	7 (14.6%)	
	Rarely	14 (29.2%)	
	Sometimes	12 (25.0%)	
Do you warn patients to avoid refractive surgery while on and 6 months after isotretinoin	Always	17 (35.4%)	
	Never	7 (14.6%)	
	Rarely	14 (29.2%)	
	Sometimes	10 (20.8%)	
Do you inform patients that while on isotretinoin they may feel discomfort while wearing contact lens, which maybe severe enough to stop them from wearing it	Always	19 (39.6%)	
	Never	6 (12.5%)	
	Rarely	7 (14.6%)	
	Sometimes	16 (33.3%)	

## Discussion

The aim of this study was to evaluate the knowledge and attitudes of dermatologists regarding ocular effects following isotretinoin prescribing in the Aseer region, Saudi Arabia. A total of 48 participants were included in the survey. In this study, the most commonly perceived complications of using isotretinoin were dry eye, contact lens intolerance, decrease in dark adaptation, ectopia lenstis and retinoblastoma. Indeed, isotretinoin is proven to be safe and effective even in young age. Early short-term use of isotretinoin can reduce the incidence of acne and also secondary acne symptoms. A study involving 90 children aged nine to 18 years with acne found that early use of isotretinoin prevents future acne lesions and post-acne scars. The study divided patients into three groups (A, B, C) based on age that were followed up for one to eight years. The results showed that 73.33% of children in Group C experienced acne scars and post-acne hyperpigmentation [22]. Elshafie et al. [21] reported that among 103 Egyptian dermatologists, 92.8% of them sometimes prescribed isotretinoin alone for moderate to severe nodulo-cystic acne, 4.5% rarely prescribed it (only for a case with primary comedone), and 2.7% always prescribed the drug for all acne types. With regard to knowledge of ocular side effects, 94.6% of dermatologists reported their knowledge and 5.4% did not know about them [23].

We found that 87.5% of the studied dermatologists thought that they can prescribe isotretinoin if the patient had recently undergone refractive surgery, 62.5% always tell patients about the side effects of this medication, three-quarters did not think that patients should have an ophthalmologic examination before prescribing isotretinoin and they rarely refer them for one, one-quarter prescribe eye lubricants, one-third always ask patients about recent refractive surgery before prescribing isotretinoin, one-third warn their patients to avoid refractive surgery during and up to six months after isotretinoin use and around two-fifths inform patients that when taking isotretinoin they may feel discomfort while wearing contact lens. These findings highlight the inadequate knowledge and practice of dermatologists in the Aseer region with regard to isotretinoin prescribing. On the other hand, a higher level of knowledge and proper practice was reported in a study that surveyed 150 dermatologists in Saudi Arabia regarding dry eye disease, refractive surgery and contact lens use when prescribing isotretinoin, with 48.3% always prescribing isotretinoin. Most dermatologists were aware of the drug's ocular side effects, but few asked about recent refractive surgery. Most advised against refractive surgery for at least six months after taking a course of isotretinoin. Most dermatologists also warned patients about contact lens intolerance [23]. Another study was conducted in Saudi Arabia to assess the perspective of patients regarding isotretinoin in order to evaluate their awareness of side effects and understanding of the practices of the treating physicians: approximately 71.2% were not advised to see an ophthalmologist for screening, 57.5% were unaware of contact lens intolerance, 67.9% were aware of the ocular side effects and 52.2% were not provided with sufficient information [24]. Alshaalan [25] investigated the knowledge of isotretinoin use and side effects among female acne patients in Saudi Arabia. A cross-sectional survey of 768 participants found that most were aware of dry mouth and lips (84.5%), teratogenicity (68.2%) and headache (44.8%). However, 60% belonged to the low knowledge category.

Strengths and limitations

To the best of our knowledge, this is the first study to assess dermatologists’ knowledge about isotretinoin-related side effects in the Aseer region, Saudi Arabia. However, this study does have several limitations. The sampling methods employed (convenience and snowball sampling), although practical, may introduce some degree of sampling bias. Participants who are more accessible or possess stronger opinions on the topic may be disproportionately represented, potentially affecting the generalizability of the findings. Self-selection bias is another concern, given that the respondents voluntarily participated in the online survey. Those with a heightened interest in or knowledge about isotretinoin-related ocular side effects may be more inclined to respond, potentially introducing bias.

## Conclusions

The findings of the survey give valuable insights into dermatologists' perceptions and practices regarding the ocular side effects of isotretinoin, a medication commonly used in dermatology. The findings revealed a unanimous acknowledgment among dermatologists that isotretinoin can lead to dry eye, highlighting a critical awareness of this side effect. Additionally, a substantial percentage recognized its potential to cause contact lens intolerance and a decrease in dark adaptation. It is noteworthy that a smaller fraction believed it could lead to ectopia lentis and retinoblastoma, indicating a range of awareness levels. In terms of attitude, a majority of dermatologists expressed cautiousness by refraining from recommending isotretinoin shortly after refractive surgery and consistently informing patients about ocular side effects. However, it is notable that a considerable proportion did not consider referring patients for ophthalmic examinations before initiating isotretinoin, suggesting a potential area for improvement in patient safety practices. The survey also unveiled diverse practices in prescribing lubricant eye drops and discussing refractive surgery avoidance with patients. These findings underscore the importance of ongoing education and collaboration between dermatologists and ophthalmologists to ensure comprehensive care and minimize ocular side effects associated with isotretinoin use.

## Appendices

**Table 3:** Dermatologists’ Knowledge and Attitude Toward Isotretinoin related Ocular Side Effects in Aseer, Saudi Arabia (Study questionnaire)

1- Do you consent to participate in this research?
- Yes - No
2- Gender
- Male - Female
3- Age:
- 25-30 - 31-40 - 41-50 - 51-60 - Older than 60
4- Qualification
- Resident - Specialist - Consultant
5- Health institution
- Ministry of health hospital - Military hospital - Private clinics - University
6- Do you know about the ocular side effects of isotretinoin?
- Yes - No
7- Which of the following are ocular side effect of isotretinoin?
- Dry eye - Contact lens intolerance - Refractive surgery precaution - Retinoblastoma - Glaucoma - Decrease dark adaptation - Ectopia lentis
8- Starting an isotretinoin course is not recommended if the patient had recently undergone refractive surgery?
- Yes - No
9- Do you inform your patients about the ocular side effects of isotretinoin?
- Always - Sometimes - Rarely - Never
10- Do you think it is important to refer the patient to do an ophthalmic exam before starting isotretinoin course?
- Yes - No
11- How often do you refer to an ophthalmologist before starting isotretinoin course?
- Always - Sometimes - Rarely - Never
12- Do you prescribe lubricant eye drops with isotretinoin:
- Always - Sometimes - Rarely
Never-
13- Do you ask patients about recent refractive surgery before starting isotretinoin:
- Always - Sometimes - Rarely - Never
14- Do you warn patients to avoid refractive surgery while on and 6 months after isotretinoin?
- Always - Sometimes - Rarely - Never
